# Supplementary figures and images for: Studies on the hysteresis of trunk muscles—Muscular specificities must be taken into account
Source: PLoS One. 2024 Dec 17;19(12):e0315813. doi: 10.1371/journal.pone.0315813 (PMC11651586; doi:10.1371/journal.pone.0315813)

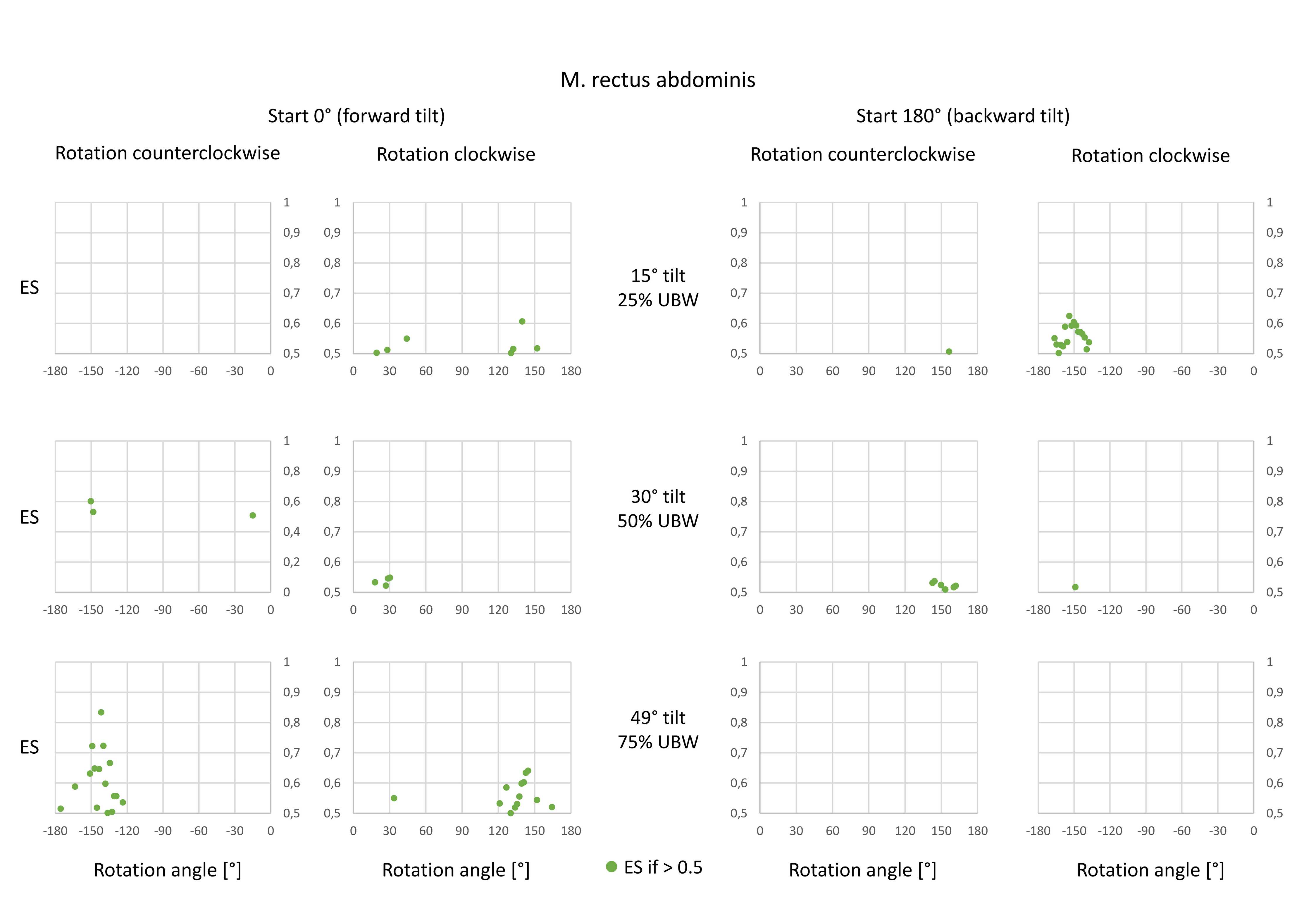

Supplement: S1 Fig — X-axes represent the respective rotation angle for the first half-phase of the complete 360° rotation. UBW: upper body weight. (TIF) [file pone.0315813.s001.tif]

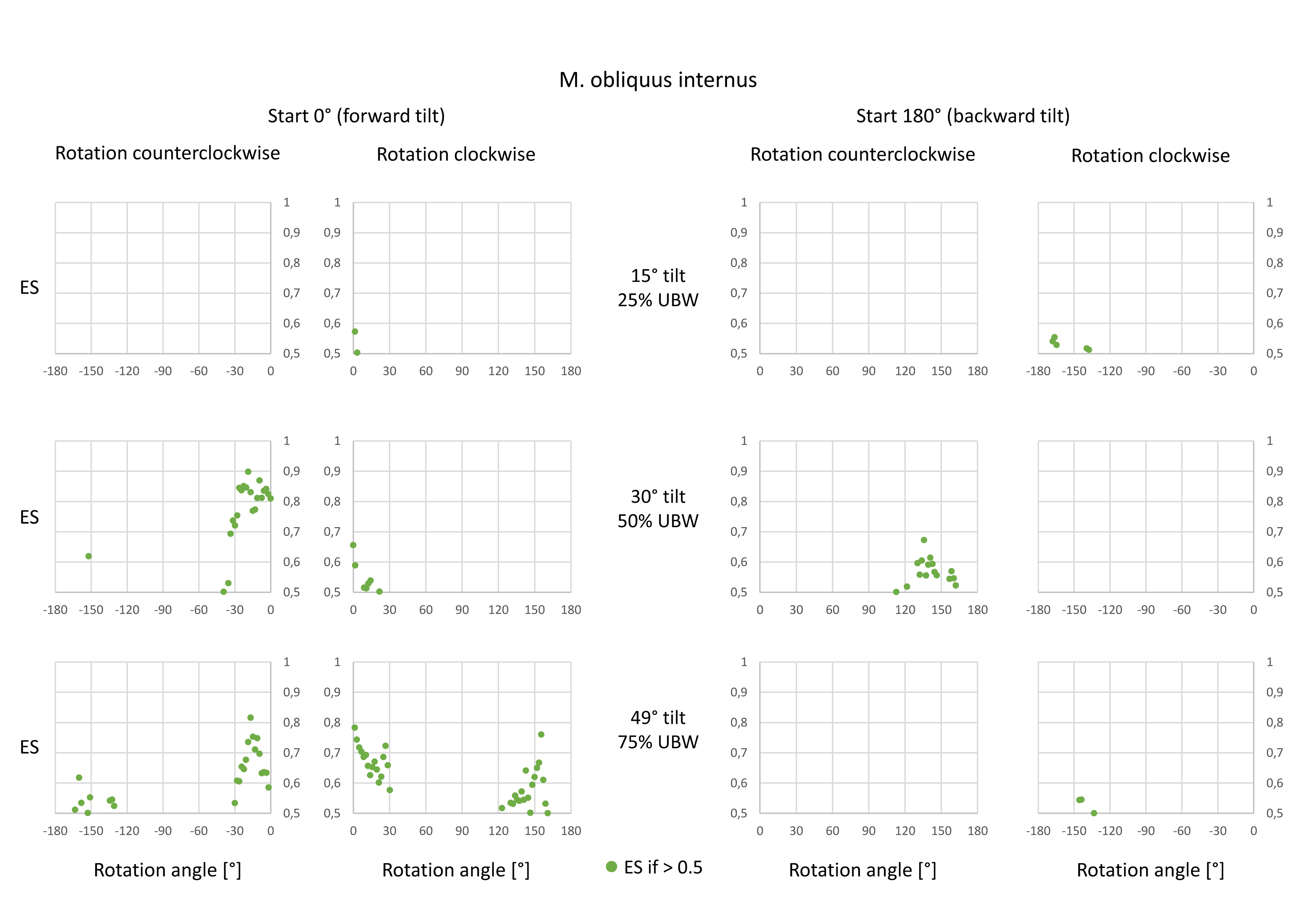

Supplement: S2 Fig — X-axes represent the respective rotation angle for the first half-phase of the complete 360° rotation. UBW: upper body weight. (TIF) [file pone.0315813.s002.tif]

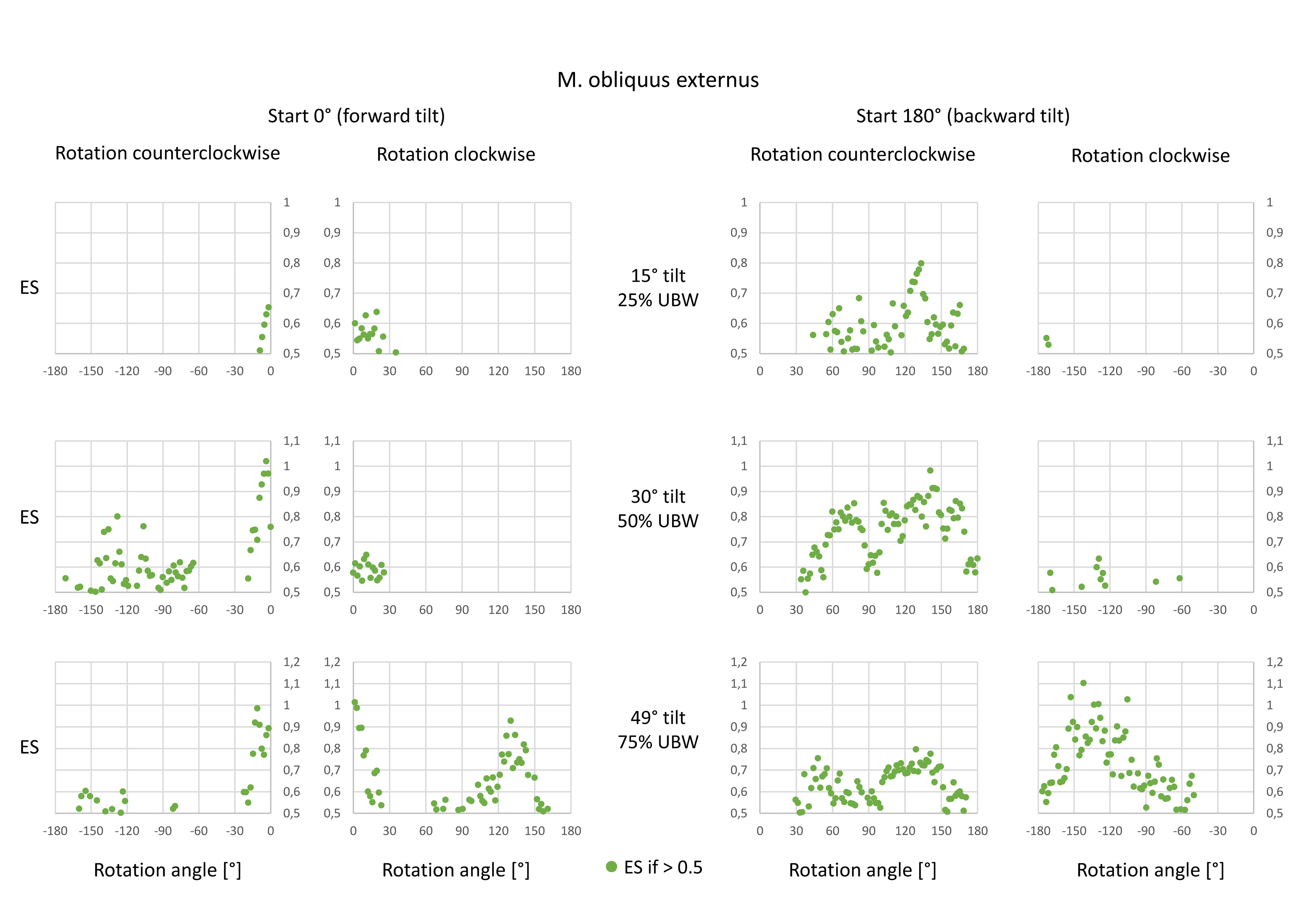

Supplement: S3 Fig — X-axes represent the respective rotation angle for the first half-phase of the complete 360° rotation. UBW: upper body weight. (TIF) [file pone.0315813.s003.tif]

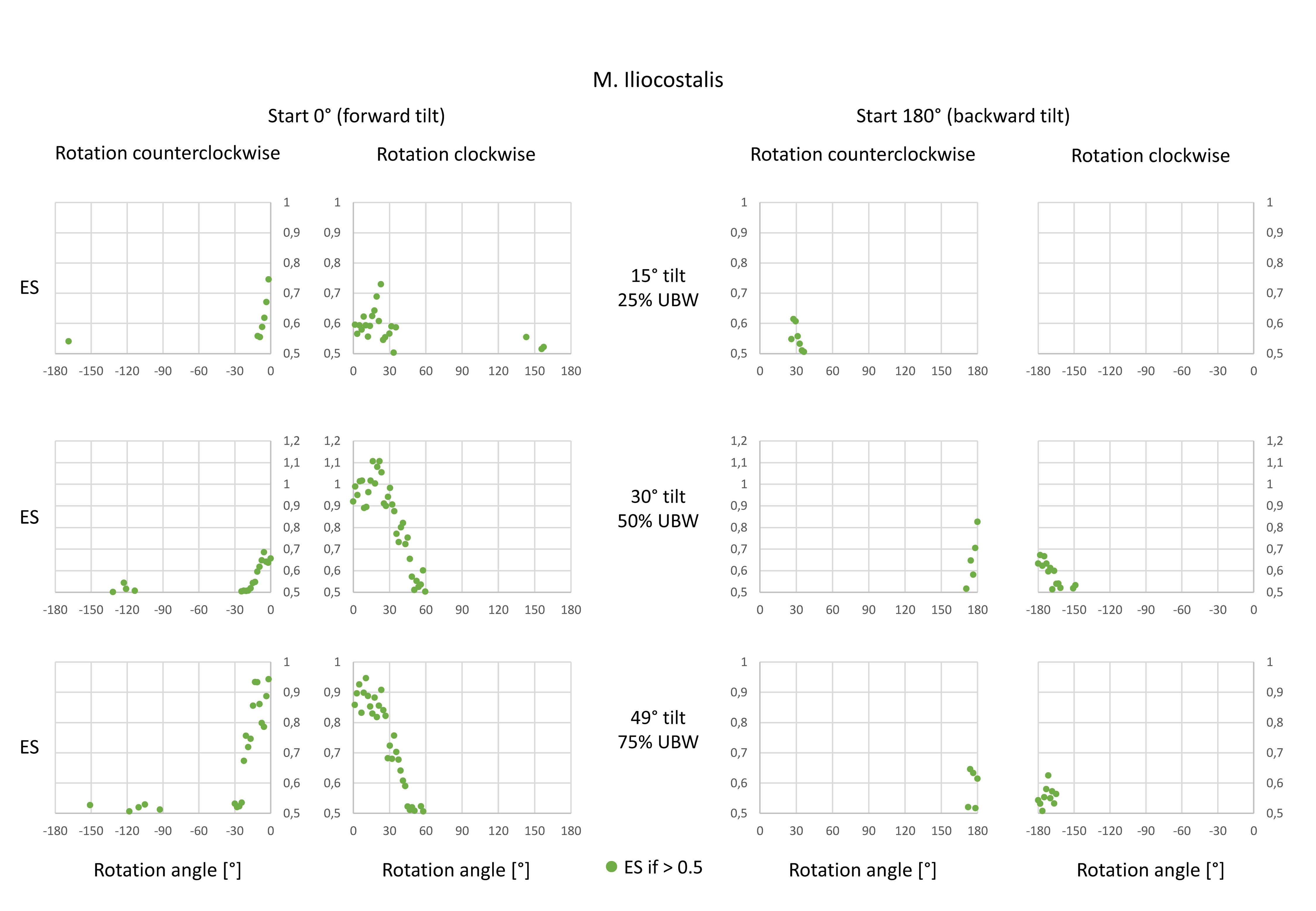

Supplement: S4 Fig — X-axes represent the respective rotation angle for the first half-phase of the complete 360° rotation. UBW: upper body weight. (TIF) [file pone.0315813.s004.tif]

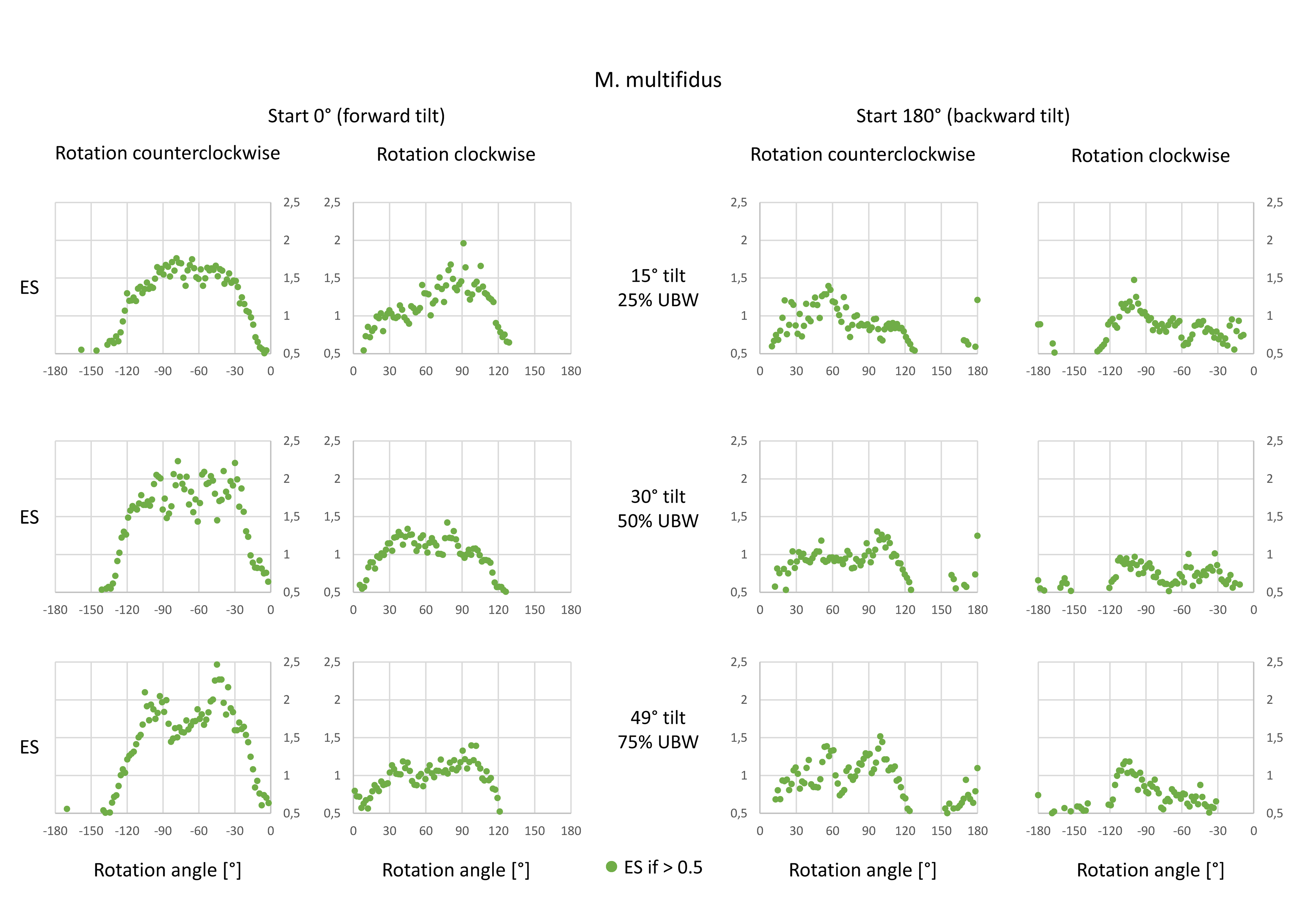

Supplement: S5 Fig — X-axes represent the respective rotation angle for the first half-phase of the complete 360° rotation. UBW: upper body weight. (TIF) [file pone.0315813.s005.tif]

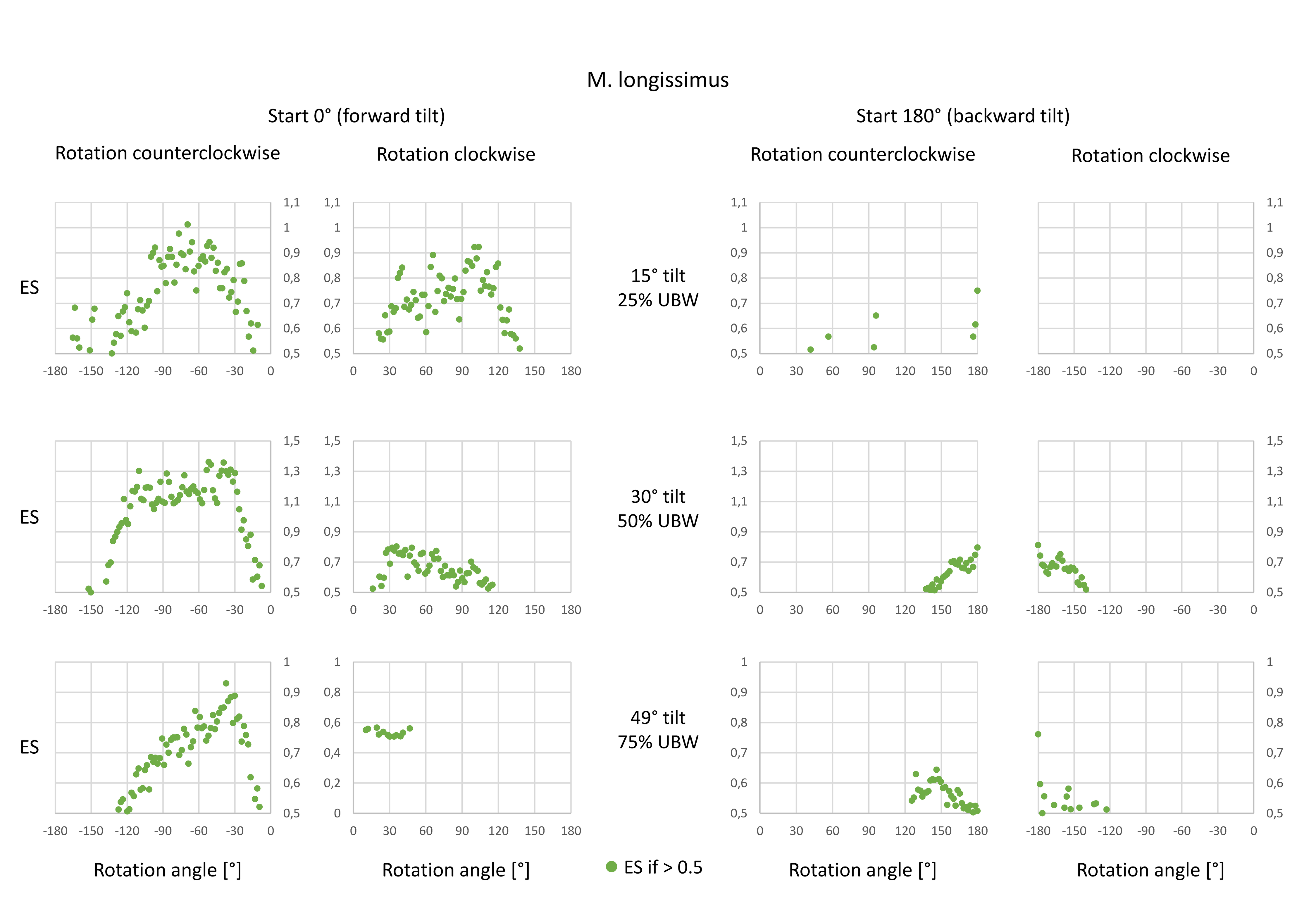

Supplement: S6 Fig — X-axes represent the respective rotation angle for the first half-phase of the complete 360° rotation. UBW: upper body weight. (TIF) [file pone.0315813.s006.tif]
